# Einschätzung des Risikos für arzneimittelbezogene Probleme bei Pflegeheimbewohner*innen: Eine retrospektive Analyse von Routinedaten

**DOI:** 10.1007/s00391-022-02152-1

**Published:** 2022-12-28

**Authors:** Fabian Graeb, Christian Jüttner, Bianca Berger, Reinhold Wolke, Jana Reißner, Gundula Essig, Petra Reiber

**Affiliations:** 1 Fakultät Soziale Arbeit, Bildung und Pflege, Institut für Gesundheits- und Pflegewissenschaften, Hochschule Esslingen, Flandernstr. 101, 73732 Esslingen, Deutschland; 2 Krankenhausapotheke, Klinikum Esslingen, Esslingen, Deutschland

**Keywords:** MERIS, Hospitalisierung, Sturz, Gewichtsverlust, Morbidität, MERIS, Hospitalization, Fall, Weight loss, Morbidity

## Abstract

**Hintergrund:**

Polypharmazie und arzneimittelbezogene Probleme (AbP) stellen eine große Herausforderung bei der Betreuung und Behandlung von Pflegeheimbewohner*innen dar. Viele Interventionsstudien zeigen enttäuschende Ergebnisse, wodurch sich die Frage stellt, ob dies auch an der Auswahl der Zielparameter liegen könnte.

**Material und Methoden:**

Mithilfe eines Routinedatensatzes aus 6 Langzeitpflegeeinrichtungen soll retrospektiv geprüft werden, ob der kürzlich validierte Medication Risk Score (MERIS) geeignet ist, in einer Population von Pflegeheimbewohner*innen eine Risikoeinschätzung vorzunehmen. Geprüft wurden Assoziationen zwischen MERIS und den abhängigen Variablen Klinikeinweisungen und Stürze über 12 Monate sowie ein Gewichtsverlust ≥ 5 % pro 3 Monate.

**Ergebnisse:**

Von 495 Bewohner*innen weisen gemäß MERIS 38,6 % (*n* = 191) eine hohes Risiko für AbP auf. Eine univariate Regressionsanalyse erbrachte bei hohem MERIS ein signifikant erhöhtes Risiko für Krankenhauseinweisungen (OR 2,2; *p* < 0,001) und einen Gewichtsverlust ≥ 5 % pro 3 Monate (OR 1,95; *p* = 0,041), jedoch keine signifikante Assoziation mit Stürzen. In der multivariaten Regression steigt das Risiko für eine Krankenhauseinweisung mit einem Diabetes mellitus (OR 1,88; *p* = 0,004), erfolgtem Sturz im selben Zeitraum (OR 1,91; *p* = 0,001), positivem MERIS (OR 1,75; *p* = 0,006) und sinkt bei stabileren Gewichtsverläufen (OR 0,88; *p* = 0,004).

**Diskussion:**

Die Ergebnisse deuten das Potenzial des Scores für zukünftige Forschungsprojekte und die individuelle Risikoeinschätzung an. Aufgrund der Einschränkungen bei retrospektiven Sekundäranalysen bedarf es aber weiterer Studien.

## Abstract (en)

**Background:**

Polypharmacy and drug-related problems are major challenges in the care and treatment of nursing home residents. Many interventional studies showed disappointing results, which lead to the question if this could also be due to the selection of the target parameters of these studies.

**Material and methods:**

A routine data set from six long-term care facilities was retrospectively analyzed. The question is if the recently validated medication risk score (MERIS) is suitable for carrying out a risk assessment in a population of nursing home residents. Associations between MERIS and the dependent variables hospital admissions and falls over 12 months and a weight loss of ≥ 5% over 3 months were examined.

**Results:**

Out of 495 residents 38.6% (*n* = 191) have a high risk of drug-related problems according to MERIS. A univariate regression analysis showed a significantly increased risk of hospital admissions (OR 2.2; *p* < 0.001) and weight loss of ≥ 5% (OR 1.95; *p* = 0.041) with high MERIS, but no significant association with falls. In the multivariate regression the risk of hospitalization was increased by diabetes mellitus (OR 1.88; *p* = 0.004), falls in the same period (OR 1.91; *p* = 0.001), positive MERIS (OR 1.75; *p* = 0.006) and decreased with stable weight (OR 0.88; *p* = 0.004).

**Conclusion:**

The results indicate the potential of the score for future research projects and individual risk assessment; however, due to the limitations of retrospective secondary analyses further studies are required.

## Hintergrund

Die steigende Lebenserwartung führt zu einer immer größeren Anzahl an Menschen im höheren Alter. Dies ist auch mit einer zunehmenden Morbidität assoziiert, was in der Konsequenz zu einem Mehr an medizinischer Behandlung und folgerichtig eher zu einer Polypharmazie führt [3, 7, 9], wovon Pflegeheimbewohner*innen überproportional häufig betroffen sind [13]. Die Definition einer Polypharmazie ist nicht einheitlich, wobei in vielen Arbeiten 5 oder mehr Arzneistoffe am Tag benannt werden [9]. Wenig überraschend steigen mit der Anzahl der einzunehmenden Medikamente auch die Risiken für arzneimittelbezogene Probleme (AbP). Tatsächlich lassen sich bei älteren Menschen relativ häufig Medikationsfehler finden [17], es kommt zum häufigeren Verschreiben von im Alter potenziell inadäquaten Medikamenten (PIM; [14]) und zu mit Polypharmazie assoziierten Folgen wie Klinikeinweisungen [11]. Etwa 3–9 % aller Krankenhauseinweisungen lassen sich auf solche AbP zurückführen, wovon die Hälfte wiederum als vermeidbar betrachtet werden kann [1]. Ein wesentlicher Grund für diese lange bekannte Problematik, speziell in der stationären Langzeitpflege, kann an massiven strukturellen Defiziten bei Kommunikation und Kooperation zwischen Pflege, (Haus‑)Ärzt*innen und Apotheken festgemacht werden [5]. Als Folge kommt es u. a. zu selten zu gezielten Absprachen und Informationsweitergaben hinsichtlich unerwünschter Arzneimittelwirkungen (UAW) sowie festgestellter Verordnungs- oder Einnahmefehler.

In groß angelegten Forschungsprojekten wurde versucht, diese Herausforderung in der Langzeitpflege zu bearbeiten und so die Sicherheit der Arzneimitteltherapie (AMTS) für diese vulnerable Gruppe zu erhöhen. In der Cluster-randomisierten kontrollierten HIOPP-3-Studie (Hausärztliche Initiative zur Optimierung der Patientensicherheit bei Polypharmazie) wurden beispielsweise Medikamentenreviews, Fortbildungen und Change-Management-Seminare für Hausärzt*innen, Apotheker*innen und Pflegekräfte durchgeführt. Dies hatte aber keinen signifikanten Effekt auf die Verordnung von PIM oder Neuroleptika sowie Sturzinzidenz, Hospitalisierungen, Lebensqualität oder Gesundheitskosten [10]. Auch eine systematische Metaanalyse von 8 randomisierten kontrollierten Studien konnte für Medikationsreviews keine signifikant positiven Effekte auf das Hospitalisierungs- und Mortalitätsrisiko nachweisen [19].

Die ernüchternden Ergebnisse vieler dieser groß angelegten Interventionsstudien verweisen auf verschiedene Herausforderungen. Diese sind z. T. systemimmanent, insofern, dass die räumliche Trennung von für die Bewohner*innen zuständigen Pflegekräften, den diversen zuständigen Hausärzt*innen und beliefernden Apotheken bislang auch durch keine digitale Vernetzung aufgelöst werden konnte. Zudem ist unklar, inwieweit sich in einer Gruppe polymorbider Patient*innen tatsächlich effektiv Medikamente einsparen oder medikamentöse Therapien minimieren lassen. Gleichermaßen kann davon ausgegangen werden, dass nicht jede Polypharmazie im selben Maße problematische Nebeneffekte wie Wechselwirkungen zeigt und damit auch nicht immer durch das Einsparen von Verordnungen positive Effekte auf z. B. das Hospitalisierungsrisiko zu erwarten sind.

Aus diesem Grund entstand die Idee, sich bei der Entwicklung einer Intervention an Personen zu orientieren, die aufgrund der komplexen Medikation ein individuell erhöhtes Risiko für AbP aufweisen. Eine Möglichkeit, diese Gefährdung anhand eines Scores einzuschätzen, bietet der von Saedder et al. entwickelte Medication Risk Score (MERIS) [16]. Dieser wurde erst kürzlich von Høj et al. anhand einer Kohorte Patient*innen mit Polypharmazie validiert [8]. Nun sollte in einer ersten Datenanalyse ermittelt werden, ob der MERIS auch in der Zielgruppe Pflegeheimbewohner*innen aussagekräftig hinsichtlich eines erhöhten Risikos für Klinikaufenthalte, Stürze und Gewichtsverluste sein und zukünftig als Instrument für die Detektion besonders vulnerabler Patient*innen dienen könnte.

## Methodik

Für diese Studie wurde ein vorliegender Datensatz aus der stationären Langzeitpflege für eine retrospektive Sekundäranalyse herangezogen. Aus insgesamt 6 Pflegeeinrichtungen eines Altenhilfeträgers wurden im Rahmen des von der IKK classic finanzierten Projektes PEBKO von allen am 01.01.2018 in diesen Einrichtungen lebenden 567 Bewohner*innen Routinedaten erhoben. Im Verlauf sind 27 dieser Personen ausgezogen, weitere 45 in den ersten 3 Monaten des insgesamt 3‑jährigen Beobachtungszeitraums verstorben und wurden daher ausgeschlossen. Die Daten der verbliebenen 495 Bewohner*innen wurden bei Erfassung umfangreich anonymisiert, sodass Rückschlüsse auf die Person nicht mehr möglich sind. Für das Projekt PEBKO liegt ein positives ethisches Clearing des Ethikkomitees der Deutschen Gesellschaft für Pflegewissenschaft vor (Antrag-Nr. 19-009).

Der analysierte Datensatz umfasst Angaben zu Alter, Geschlecht, Erkrankungen, Pflegegrad und Medikamenten, über einen Zeitraum von 3 Jahren (01.01.2018–01.01.2021) außerdem regelmäßige Gewichtsmessungen, BMI, Angaben zu Stürzen, Schmerzen, Klinikaufenthalten, Dekubitus-Entwicklung und Versterben. Die Morbidität wurde mithilfe des Charlson-Komorbiditätsindex in der gekürzten Fassung nach Quan engeschätzt [15].

Zur Ermittlung eines AbP-Risikos wurde der MERIS herangezogen und der von Høj et al. validierte Cut-off Wert von 15 verwendet. Der Score basiert auf einer Bewertung der Nierenfunktion mithilfe der „estimated glomerular filtration rate“ (eGFR), der Medikamentenanzahl und einer Risikoeinschätzung aller eingenommen Medikamente (Risiko auf direkte Schädigung und Interaktionsrisiko). Als Hilfsmittel wurde die im Online-Supplement der Publikation hinterlegte Tabelle verwendet [8]. Als problematisch erwies sich die Einschätzung der Nierenfunktion, da in den analysierten Routinedaten keine Laborwerte enthalten sind. Nach Rücksprache mit den Pflegeeinrichtungen zeigte sich, dass sich diese Labordaten nicht nacherheben lassen. Wird von ärztlicher Seite ein Routinelabor abgenommen, dann verbleiben die Ergebnisse üblicherweise in der Praxis und werden nur in wenigen Fällen auch in der Bewohnerakte der Einrichtung abgelegt. Ersatzmäßig wurden daher die Diagnose einer Niereninsuffizienz und Angaben zum Erkrankungsstadium herangezogen. Die im MERIS definierten Grenzwerte der eGFR entsprechen dabei der Stadieneinteilung gemäß ICD-10-Katalog, sodass eine entsprechende Bewertung vorgenommen werden konnte.

Die statistische Analyse wurde mithilfe von SPSS 28 (IBM, Armonk, NY, USA) durchgeführt. Die Analyse folgt der Hypothese, dass ein hoher MERIS signifikant mit der Häufigkeit an Klinikeinweisungen, Stürzen und erheblichen Gewichtsverlusten assoziiert ist. Für den Gruppenvergleich hohes Risiko gemäß MERIS und kein hohes Risiko wurden *t*-Tests mit vorangestelltem *F*-Test (metrisch skalierte Variablen) und Chi^2^-Tests (ordinalskalierte Variablen) herangezogen. Die Effektstärke *r* wurde anhand des Korrelationskoeffizienten Pearson bzw. Phi bestimmt. Für die Zielparameter mindestens ein Klinikaufenthalt und ein Sturz innerhalb von 12 Monaten sowie ≥ 5 % Gewichtsverlust pro 3 Monate wurde zunächst eine univariate logistische Regression mit der unabhängigen Variable MERIS-Cut-off gerechnet. Bei Vorliegen einer signifikanten Assoziation wurde dann jeweils eine multivariate logistische Regressionsanalyse gerechnet. Dabei wurden die unabhängigen Variablen explorativ schrittweise vorwärts in das Modell eingeschlossen. Ein *p* < 0,05 wird als signifikant betrachtet.

## Ergebnisse

Tab. 1 beschreibt die Kohorte insgesamt und differenziert anhand der Risikoeinschätzung nach MERIS. Mit 82,9 Jahren (SD ±9,3) ist die Gruppe hochaltrig, überwiegend weiblich (68,1 %) und weist in zwei Dritteln der Fälle einen Pflegegrad von 4 bis 5 auf (66,1 %). Beide Gruppen unterscheiden sich hierbei nicht signifikant. Allerdings leiden Bewohner*innen mit einem hohen MERIS häufiger unter chronischen Erkrankungen wie arterieller Hypertonie (74,3 % vs. 55,9 %; *p* < 0,001; r = 0,186), Diabetes mellitus (35,1 % vs. 19,4 %; *p* < 0,001; r = 0,175), Niereninsuffizienz (30,4 % vs. 15,1 %; *p* < 0,001; r = 0,182), ischämischen Herzkrankheiten (31,4 % vs. 13,5 %; *p* < 0,001; r = 0,217) und affektiven Störungen (24,6 % vs. 13,5; *p* = 0,002; r = 0,142). Eine Demenz liegt dagegen seltener vor (38,7 % vs. 52,3 %; *p* = 0,003; r = 0,132). Bewohner*innen mit hohem MERIS büßen in den ersten 3 Monaten signifikant mehr Gewicht ein (−0,6 kg vs. −0,2 kg; *p* = 0,047; r = 0,109), verlieren häufiger mindestens 5 % ihres Körpergewichts (11,5 % vs. 6,3 %; *p* = 0,038; r = 0,093) und werden häufiger stationär im Krankenhaus behandelt (45,0 % vs. 28,9 %; *p* < 0,001; r = 0,164). Nicht signifikant unterscheiden sich die Gruppen hinsichtlich Dauer der Klinikaufenthalte, Morbidität gemäß Charlson-Komorbiditätsindex, Gewicht und BMI. Tendenziell häufiger, aber ohne signifikanten Unterschied kommt es zu Stürzen, Dekubitus und Versterben.

**Table 1:** 

		Alle		MERIS, kein hohes Risiko		MERIS, hohes Risiko		*p*^a^	*r*^b^
		(*n* = 495)		(*n* = 304; 61,4 %)		(*n* = 191; 38,6 %)			
Alter	M (±SD)	82,9	(±9,3)	82,7	(±10,1)	83,3	(±7,9)	0,590	–
Geschlecht, weiblich	% (*n*)	68,1	(337)	68,8	(209)	67,0	(128)	0,687	–
Hoher Pflegegrad (4–5)	% (*n*)	66,1	(327)	65,8	(200)	66,5	(127)	0,827	–
*Chronische Erkrankungen*									
Art. Hypertonie	% (*n*)	63,0	(312)	55,9	(170)	74,3	(142)	< 0,001	0,186
Demenz	% (*n*)	47,1	(233)	52,3	(159)	38,7	(74)	0,003	0,132
Diabetes mellitus	% (*n*)	25,5	(126)	19,4	(59)	35,1	(67)	< 0,001	0,175
Niereninsuffizienz	% (*n*)	21,0	(104)	15,1	(46)	30,4	(58)	< 0,001	0,182
Ischämische Herzkrankheit	% (*n*)	20,4	(101)	13,5	(41)	31,4	(60)	< 0,001	0,217
Affektive Störungen	% (*n*)	17,8	(88)	13,5	(41)	24,6	(47)	0,002	0,142
Arthropathien	% (*n*)	18,4	(91)	16,4	(50)	21,5	(41)	0,161	–
Z. n. Apoplex	% (*n*)	16,2	(43)	16,1	(49)	16,2	(31)	0,974	–
Osteopathien	% (*n*)	15,8	(78)	12,8	(39)	20,4	(39)	0,024	0,101
Anämien	% (*n*)	14,7	(73)	12,5	(38)	18,3	(35)	0,075	–
Sturzneigung	% (*n*)	13,3	(66)	9,2	(28)	19,9	(38)	0,001	0,153
Herzinsuffizienz	% (*n*)	12,9	(64)	9,5	(29)	18,3	(35)	0,005	0,127
Tumorerkrankungen	% (*n*)	9,7	(48)	8,9	(27)	11,0	(21)	0,439	–
Morbus Parkinson	% (*n*)	8,3	(41)	4,9	(15)	13,6	(26)	0,001	0,153
Morbiditätsindex nach Charlson	M (±SD)	2,4	(±1,9)	2,4	(±2,0)	2,5	(±1,8)	0,473	–
BMI in kg/m^2^	M (±SD)	25,9	(±5,1)	25,6	(±5,0)	26,3	(±5,2)	0,157	–
Gewicht in kg	M (±SD)	69,3	(±17,1)	68,3	(±16,7)	70,9	(±17,7)	0,100	–
Gewichtsveränderungen pro 3 Monate in kg^c^	M (±SD)	−0,4	(±2,3)	−0,2	(±2,0)	−0,6	(±2,6)	0,047	0,109
Gewichtsveränderungen pro 3 Monate in %^c^	M (±SD)	−0,5	(±3,5)	−0,3	(±3,1)	−0,9	(±4,0)	0,106	–
Gewichtsverlust ≥ 5 % in 3 Monaten	% (*n*)	8,3	(41)	6,3	(19)	11,5	(22)	0,038	0,093
Anzahl d. Medikamente	M (±SD)	10,8	(±4,7)	8,0	(±2,8)	15,3	(±3,3)	< 0,001	0,782
MERIS	M (±SD)	12,2	(±6,2)	8,0	(±3,8)	18,8	(±2,6)	< 0,001	0,862
Mind. 1 Krankenhauseinweisung in 12 Monaten	% (*n*)	35,2	(174)	28,9	(88)	45,0	(86)	< 0,001	0,164
KH Nächte 12 Monate^d^	M (±SD)	14,1	(±15,5)	14,2	(±17,2)	13,9	(±13,7)	0,157	–
Mind. 1 Sturz 12 Monate	% (*n*)	48,5	(240)	47,4	(144)	50,3	(96)	0,531	–
Mind. 1 Dekubitus 12 Monate	% (*n*)	21,8	(108)	20,4	(62)	24,1	(46)	0,333	–
Mortalität 12 Monate	% (*n*)	17,0	(84)	16,1	(49)	18,3	(35)	0,524	–
Mortalität 36 Monate	% (*n*)	55,2	(273)	52,3	(159)	59,7	(114)	0,108	–

*KH* Krankenhaus, *M* arithmetisches Mittel, *SD* Standardabweichung

^a^ Signifikanz gemäß Chi^2^- bzw. *t*-Test

^b^ Effektstärke Phi bzw. Korrelationskoeffizient nach Pearson

^c^ *n* = 491/301/190

^d^ Nur Bewohner*innen mit Klinikaufenthalt *n* = 174/88/86

### Regressionsanalysen

Die Frage ist nun, ob sich signifikante Assoziationen zwischen einem hohen Risiko gemäß MERIS und den Outcome-Variablen Klinikaufenthalt, Sturz und Gewichtsverlust ≥ 5 % in 3 Monaten zeigen (Tab. 2). Eine univariate Regressionsanalyse erbrachte bei hohem MERIS ein signifikant erhöhtes Risiko für Krankenhauseinweisungen (OR 2,2; *p* < 0,001) und einen Gewichtsverlust ≥ 5 % (OR 1,95; *p* = 0,041), jedoch keine signifikante Assoziation mit Stürzen.

**Table 2:** 

Abhängige Variable	OR	KI (95 %)	*p*
Mind. 1 Krankenhauseinweisung in 12 Monaten^a^	2,010	1,378–2,933	< 0,001
Sturz 12 Monate^b^	1,123	0,782–1,613	0,531
≥ 5 % Gewichtsverlust/3 Monate^c^	1,953	1,027–3,713	0,041

*KI* Konfidenzintervall, *OR* „odds ratio“

^a^ (Chi^2^(1) = 13,185; *p* < 0,001; f^2^ = 0,193; *n* = 495)

^b^ (Chi^2^(1) = 0,393; *p* < 0,531; f^2^ = 0,032; *n* = 495)

^c^ (Chi^2^(1) = 4,165; *p* = 0,041; f^2^ = 0,139; *n* = 495)

Anschließend wurden unabhängige Variablen jeweils schrittweise vorwärts in eine multivariate logistische Regressionsanalyse für die beiden abhängigen Variablen Krankenhauseinweisung und Gewichtsverlust ≥ 5 % pro 3 Monate eingeschlossen (Tab. 3). Getestet wurden die Variablen Alter, Geschlecht, hoher Pflegegrad, Morbiditätsindex, Gewichtsveränderungen pro 3 Monate (nur im Modell Krankenhauseinweisung), Sturz, chronische Erkrankungen, Krankenhauseinweisungen pro 6 Monate (nur im Modell Gewichtsverlust ≥ 5 %), BMI und erhöhtes Risiko laut MERIS-Cut-off. Das Risiko für eine Krankenhauseinweisung steigt mit Diabetes mellitus (OR 1,88; *p* = 0,004), erfolgtem Sturz (OR 1,91; *p* = 0,001), hohem MERIS (OR 1,75; *p* = 0,006) und sinkt bei positiveren Gewichtsveränderungen in den ersten 3 Monaten (OR 0,88; *p* = 0,004). Das Risiko, einen Gewichtsverlust ≥ 5 % in den ersten 3 Monaten zu erleiden, ist signifikant erhöht bei hohem Pflegegrad 4–5 (OR 1,21; *p* = 0,007) und mindestens einer Klinikeinweisung in den ersten 6 Monaten (OR 2,95; *p* = 0,002). Das Risiko ist dagegen signifikant reduziert bei höherem BMI (OR 0,92; *p* = 0,018) und vorliegender Demenzerkrankung (OR 0,41; *p* = 0,014). Keine signifikante Assoziation besteht hinsichtlich eines hohen MERIS.

**Table 3:** 

	OR	KI (95 %)	P
*Mind. 1 Krankenhauseinweisung in 12 Monaten*^a^			
Gewichtsveränderungen 3 Monate in kg	0,881	0,808–0,960	0,004
Diabetes mellitus	1,881	1,217–1,909	0,004
Sturz 12 Monate	1,913	1,297–2,822	0,001
Risiko laut MERIS-Cut-off	1,748	1,177–2,595	0,006
*Gewichtsverlust ≥* *5* *% pro 3 Monate*^b^			
Hoher Pflegegrad 4–5	1,205	1,386–8,037	0,007
BMI, T_0_	0,916	0,851–0,985	0,018
Demenzerkrankung	0,413	0,203–0,839	0,014
Krankenhauseinweisungen 6 Monate	2,952	1,508–5,779	0,002

*BMI T*_*0*_ initialer Body-Mass-Index, *KI* Konfidenzintervall, *OR* „odds ratio“

^a^ (Chi^2^(4) = 39,235; *p* < 0,001; f^2^ = 0,344; *n* = 491)

^b^ (Chi^2^(4) = 28,122; *p* < 0,001; f^2^ = 0,381; *n* = 495)

## Diskussion

Der MERIS als Einschätzungsinstrument zur Darstellung eines erhöhten AbP-Risikos lässt sich auf Routinedaten der Altenpflege anwenden. Im Ergebnis zeigt sich ein ähnlich hoher Anteil an Personen mit einem entsprechenden Risiko wie in der Validierungsstudie von Høj et al. [8]. Die möglichen Assoziationen mit den Outcome-Parametern Klinikeinweisungen, Gewichtsverlust ≥ 5 % und Sturz stellen sich jedoch unterschiedlich dar. Es zeigt sich zwar, dass die Bewohner*innen mit hohem Risiko laut MERIS häufiger unter den verschiedenen chronischen Erkrankungen leiden und auch eher mindestens einen Klinikaufenthalt innerhalb von 12 Monaten aufweisen. Die multivariate Regressionsanalyse ergibt dann auch eine dementsprechende signifikante Risikoerhöhung. Unklar bleibt aber, ob diese Assoziation tatsächlich in der Medikation begründet liegt oder in den Erkrankungen. Während die Sturzhäufigkeit nicht mit einem hohen MERIS assoziiert ist, kommt es häufiger zu einem Gewichtsverlust ≥ 5 % des Körpergewichtes innerhalb von 3 Monaten. Dies überrascht kaum, da eine Polypharmazie mit Mangelernährung assoziiert ist [12]. Es sollte dabei beachtet werden, dass hier allein der Mangelernährungsparameter Gewichtsverlust ≥ 5 % pro 3 Monate gewählt wurde, um einen Effekt zu untersuchen, da der verwendete Datensatz keine Daten zu Muskelstatus, Inflammation oder verändertem Nährstoffbedarf und veränderter Nahrungszufuhr enthält. Diese Informationen wären aber erforderlich, um eine Mangelernährung entsprechend den aktuellen Kriterien der Global Leadership Initiative on Malnutrition (GLIM) zu definieren [4]. Während die univariate Regressionsanalyse eine signifikante Assoziation des Gewichtsverlustes mit einem erhöhtem Risiko gemäß MERIS zeigt, ist der Score nicht Teil des multivariaten Regressionsmodells. Allerdings steigt das Risiko für diesen Gewichtsverlust auch mit Klinikaufenthalten, was sich auch schon in früheren Erhebungen mit Daten aus der Altenpflege aufzeigen ließ [6]. Wenn nun aber über eine Verbesserung der medikamentösen Therapie Klinikeinweisungen vermieden werden könnten, gibt es möglicherwiese auch weniger Gewichtsverluste. Daher erscheint es sinnvoll, in der geplanten Interventionsstudie den Gewichtsverlust dennoch als sekundären Outcome-Parameter im Blick zu haben. Überraschend erscheint die Assoziation einer Demenz mit einem geringeren Risiko für einen Gewichtsverlust ≥ 5 %. Erklären lässt sich diese mit einem vermutlich bereits initial häufig reduzierten Ernährungsstatus.

Die durchschnittliche Anzahl der einzunehmenden Medikamente ist mit 10,8 (±4,7) hoch, selbst in der Gruppe mit niedrigem MERIS liegt diese noch bei 8,0 (±2,8). Bei Specka et al. hingegen nimmt eine Population Pflegeheimbewohner*innen im Raum Essen im Mittel 7 Medikamente ein. In dieser Erhebung wiesen 76,3 % ein klinisch relevantes Interaktionsmuster auf, basierend auf einer Analyse mit der „Clinical-Decision-Support-Software“(CDSS)-Onlinedatenbank [18]. Gerade diese Interaktionen zwischen vielen Wirkstoffen erschwert die Einschätzung des individuellen Risiko bei einer Polypharmazie. Mithilfe des MERIS kann nicht nur das Interaktionsrisiko bewertet werden, sondern auch das Risiko der Medikation für Schädigungen, das sich dann in UAW manifestiert. Vor allem bei älteren Personen ist ein dadurch erhöhtes Sturzrisiko als besonders problematisch einzustufen. So weisen auch hier die Bewohner*innen mit hohem MERIS zwar signifikant häufiger die Diagnose Sturzneigung auf, jedoch besteht kein signifikanter Unterschied zwischen den Gruppen hinsichtlich der tatsächlichen Stürze. Es gibt die Möglichkeit, das Sturzrisiko aufgrund der Medikation gezielt einzuschätzen, etwa mit der Liste *Fall-Risk-Increasing Drugs* (FRID). Zu diesen gehören u. a. Opioide, Antiepileptika, Antipsychotika und generell eine Polymedikation mit ≥ 9 verschiedenen Wirkstoffen. 16 % der Pflegeheimbewohner*innen mit FRID weisen innerhalb eines Jahres mindestens einen Sturz mit nachfolgender Hospitalisierung auf. Allerdings zeigen sich auch erhebliche Schwankungen der Sturzhäufigkeit zwischen den untersuchten Einrichtungen, was auf ein komplexes Zusammenspiel verschiedener Risikofaktoren schließen lässt [2]. Eine Fokussierung auf das Sturzrisiko macht es also evtl. erforderlich, anhand der in der FRID-Liste definierten Risikomedikation eine zusätzliche Einschätzung vorzunehmen. Andererseits wurden hier lediglich Stürze allgemein erfasst, unabhängig von der Schwere und möglichen Verletzungen mit Hospitalisierung. Diese Daten waren nicht Teil der ausgewerteten Routinedaten. Hierin liegt ohnehin die größte Limitation dieser Studie. Durch die retrospektive Analyse von Routinedaten lassen sich Lücken, wie die nichtvorliegenden Labordaten, nur schwer schließen. Inwiefern die Aussagekraft des MERIS ohne aktuelle eGFR reduziert ist, lässt sich nicht abschätzen. Allerdings stellt die Orientierung am Schweregrad der Nierenschädigung nicht nur eine pragmatische, sondern für das Setting der Langzeitpflege auch möglicherweise bessere Lösung dar. Schließlich geht es hier eher um die Einschätzung der allgemeinen und weniger der tagesaktuellen Gefährdung durch die Medikation. Gleichzeitig ist davon auszugehen, dass bei Prüfung der Medikation, etwa im Rahmen einer hausärztlichen Visite, häufig kein aktuelles Serumkreatinin vorliegt, was die Anwendung des Scores außerhalb von Studien in diesem Setting grundsätzlich erschweren würde.

## Fazit für die Praxis

- Polypharmazie und spezifische AbP sind speziell bei geriatrischen Patient*innen potenziell hochproblematisch. Eine kritische Prüfung der notwendigen Medikation kann möglicherweise dazu beitragen, diese Risiken zu verringern.
- Der MERIS könnte sowohl in der Forschung als auch in der Praxis ein geeignetes Instrument darstellen, um dieses Risiko individuell einzuschätzen. Allerdings bedarf es Interventionsstudien, um zu untersuchen, ob eine Verbesserung des Scores auch zu einem besseren Outcome beiträgt.
